# Hospitalized COVID-19 Patients with Severe Acute Respiratory Syndrome: A Population-Based Registry Analysis to Assess Clinical Findings, Pharmacological Treatment and Survival

**DOI:** 10.3390/medicina58060829

**Published:** 2022-06-19

**Authors:** Eduardo Gutiérrez-Abejón, Francisco Herrera-Gómez, M. Aránzazu Pedrosa-Naudín, Eduardo Tamayo, F. Javier Álvarez

**Affiliations:** 1 Pharmacological Big Data Laboratory, Faculty of Medicine, University of Valladolid, 47005 Valladolid, Spain; fherrerag@saludcastillayleon.es (F.H.-G.); alvarez@med.uva.es (F.J.Á.); 2 Pharmacy Directorate, Castilla y León Health Council, 47007 Valladolid, Spain; maranzazup@saludcastillayleon.es; 3 Centro de Investigación Biomédica en Red de Enfermedades Infecciosas (Group CB21/13/00051), Carlos III Institute of Health, 28029 Madrid, Spain; tamayo@med.uva.es; 4 Transplantation Center, Faculty of Medicine, Lausanne University Hospital & University of Lausanne, CH-1011 Lausanne, Switzerland; 5 Department of Kidney Resuscitation and Acute Purification Therapies, Complejo Asistencial de Zamora, Sanidad de Castilla y León, 49022 Zamora, Spain; 6 Group for Biomedical Research in Critical Care Medicine (BioCritic), Faculty of Medicine, University of Valladolid, 47005 Valladolid, Spain; 7 Department of Anesthesiology, Hospital Clínico Universitario de Valladolid, 47003 Valladolid, Spain; 8 Department of Surgery, Faculty of Medicine, University of Valladolid, 47005 Valladolid, Spain; 9 CEIm, Hospital Clínico Universitario de Valladolid, 47003 Valladolid, Spain

**Keywords:** SARS-CoV-2, COVID-19, severe acute respiratory syndrome, clinical findings, pharmacological treatment, survival

## Abstract

*Background and Objectives:* One of the most serious clinical outcomes in hospitalized patients with COVID-19 is severe acute respiratory syndrome (SARS). The aim is to analyze pharmacological treatment, survival and the main mortality predictors. *Materials and Methods:* A real-world data study from COVID-19-hospitalized patients with SARS from 1 March to 31 May 2020 has been carried out. Variables such as hospital length of stay, ventilation type and clinical outcomes have been taken into account. *Results:* In Castile and Leon, 14.03% of the 7307 in-hospital COVID-19 patients developed SARS, with a mortality rate of 42.53%. SARS prevalence was doubled in males compared to females, and 78.54% had an age of 65 years or more. The most commonly used medicines were antibiotics (89.27%), antimalarials (68.1%) and corticosteroids (55.9%). Survival of patients developing SARS was lower compared to patients without this complication (12 vs. 13 days). The main death predictors were disseminated intravascular coagulation (DIC) (OR: 13.87) and age (>65 years) (OR: 7.35). *Conclusions:* Patients older than 65 years who develop DIC have a higher probability of hospital death. Tocilizumab and steroids have been linked to a lower incidence of hospital death, being the main treatment for COVID-19 hospitalized patients with SARS.

## 1. Introduction

One of the most prevalent complications with the worst prognosis in patients hospitalized with coronavirus disease 2019 (COVID-19) remain severe acute respiratory syndrome (SARS) [1,2,3] and acute respiratory distress syndrome (ARDS) [4]. SARS is a viral pneumonia that is associated with high fever and other symptoms described in Table 1, mostly related to cytokine storms that are a consequence of systemic inflammatory response syndrome (SIRS) [5].

In this context, increased levels of proinflammatory cytokines have been reported, not only in the acute phase of SARS, particularly interleukin-6 (IL-6), but also in advanced stages of the disease, even in patients requiring invasive mechanical ventilation (IMV), such as IL-6, interleukin-8 (IL-8), interleukin-9 (IL-9), interleukin-17 (IL-17), interferon gamma-induced protein 10 (IP-10), monocyte chemoattractant protein 1 (MCP-1), granulocyte-colony stimulating factor (G-CSF), macrophage inflammatory protein-1-alpha (MIP-1) and chemokine ligand 5 (RANTES) [6,7].

Coagulation disorders including an exaggerated fibrinolysis into the pulmonary circulation are also considered relevant factors for the development of SARS [8]. Particularly, disseminated intravascular coagulation (DIC) triggers a rapid worsening of the patient’s state, and is associated with a high mortality rate [9].

In the clinical arena, the severity of SARS depends on the degree of hypoxemia: in worse cases, the patient may require invasive mechanical ventilation (IMV) [10], and be admitted to intensive care units (ICU) [11]. The necessity to treat the other companion alterations is a condition sine qua non.

Briefly, we present research with the intention to describe the most critical COVID-19 patients, the presence of SARS, SIRS and DIC, sensitize clinicians and target efforts all over the world to combat the 21st century pandemic [11,12].

In a previous article [3], prevalence of SARS was observed at 14.03% among hospitalized COVID-19 patients in Castile and Leon during the three first months of the pandemic (March 1st to May 31th, 2020). In this regard, the main aim of this study is to provide real-world data about in-hospital COVID-19 patients affected by SARS in our region, the largest of Spain, with 2,323,770 inhabitants and a network hospital capacity of 7141 beds spread over 14 hospitals (three regional hospitals, six general hospitals and five first-level referral hospitals). As a continuation of previously conducted research [3,13,14], this manuscript presents an analysis of patterns of drug use, survival, mortality rate and main predictors of mortality among the patients described above. These results complete the in-depth analysis of clinical conditions with worse prognosis for hospitalized COVID-19 patients, such as SARS, acute kidney injury (AKI) [13] and previous cardiovascular disease (CVD) [14].

## 2. Materials and Methods

### 2.1. Real-World Study Details

This manuscript presents an epidemiological study carried out using real-world data and the following recommendations to present observational evidence: the Reporting of studies Conducted using Observational Routinely collected health Data (RECORD) recommendations [15] and the Strengthening the Reporting of Observational Studies in Epidemiology (STROBE) standards [16]. The ethical approval was granted by CEIm Área de Valladolid Este (PI-20-1863).

SARS diagnosis was established by in-hospital treating physicians according the clinical or radiological findings described in Table 1.

Information on the patient’s management and treatment was obtained from health records stored in JIMENA, its equivalent for out-hospital data MEDORA (https://www.saludcastillayleon.es/sanidad/cm, accessed on 4 November 2021), the Basic Minimum Data Set of Hospital Discharges registry of Castile and Leon (https://pestadistico.inteligenciadegestion.mscbs.es/publicoSNS/N/rae-cmbd/rae-cmbd, accessed on 4 November 2021), and the platform collecting information on dispensation of medicines for the health system beneficiaries in Castile and Leon, CONCYLIA (http://www.saludcastillayleon.es/portalmedicamento/es/indicadoresinformes/Concylia, accessed on 4 November 2021).

### 2.2. Variables

Comorbidities as hypertension, cardiovascular disease, diabetes, chronic respiratory disease, neoplasia, autoimmune disease and chronic kidney disease, hospital stay as the hospitalization period (in days) and length of stay in ICU (in days), ventilation need, and clinical complications, as acute kidney injury (AKI), fungal and bacterial superinfection, SIRS, cardiomyopathy and DIC were considered as variables. National recommendations at the study period were used for selecting the medicines to analyze [17,18] (Supplementary Table S1). All variables were obtained according to gender and age.

### 2.3. Statistical Analysis

For a better understanding of our analysis, 15-day periods were established, for which data are expressed in percentages with 95% confidence interval (95% CI) or in medians with interquartile range (IQR). In comparisons, the Student’s *t*-test or the Mann–Whitney U test for continuous variables, and Pearson’s Chi-squared test or Fisher’s exact test for categorical variables, were used, as appropriate. The normal distribution of the data for each variable was assessed using the Kolmogorov–Smirnov and Shapiro–Wilk tests.

Furthermore, Kaplan–Meier survival analysis and comparisons of survival between groups using the log-rank test were performed. Predictors of in-hospital death were obtained using multivariate logistic regression using the forward conditional model. The variables of age (>65), gender, comorbidities, the need for ventilation, the variables related to medication use (antibiotics, antimalarials, steroids, antivirals (lopinavir-ritonavir), tocilizumab and anti-SIRS drugs) and clinical complications were included in the model. Significance level was defined at *p* ≤ 0.05. Statistical Package for the Social Sciences (SPSS) version 24.0 (IBM, Armonk, NY, USA) was used in all calculations.

## 3. Results

### 3.1. Clinical Findings

During March to May 2020, 7307 patients were hospitalized for COVID-19 in Castile and Leon and 1025 developed SARS (14.03%). Prevalence of this affectation was twice as common in male than in female (*p* = 0.004), and four out of five patients had an age of 65 years or more, with a mortality of 42.53%, which increased to 85% in the case of patients with DIC. Half of the patients had hypertension, 39.61% cardiovascular disease, 21.56% diabetes mellitus, and 16.49% chronic respiratory affectation. Overall, these patients presented during their hospital stay acute kidney injury (18.73%), and fungal (5.37%) and bacterial infections (4.39%) (Table 2). 

**Figure 1:**
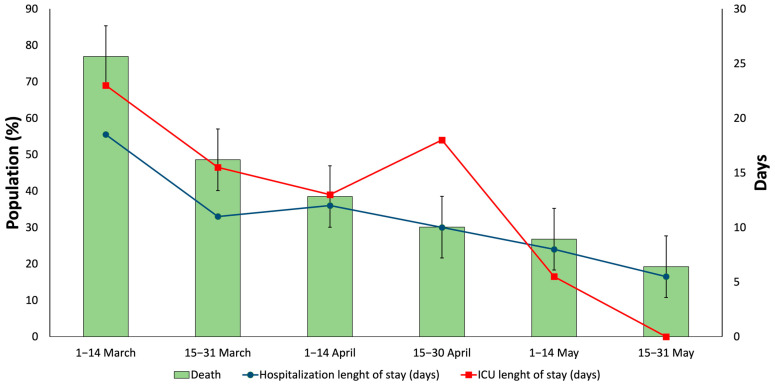
Death and hospital length of stay corresponding to the in-hospital COVID-19 patients with severe acute respiratory syndrome in Castile and Leon (Spain) (1 March–31 May 2020).

IMV was used four times more than only conventional oxygen delivery or noninvasive positive pressure ventilation (NPPV). In addition, at the beginning, mortality was 76.92%, which decreased to a quarter at the end of May. In addition, at the beginning, medians of hospital stay and ICU stay were, respectively, 18 and 23 days, which then decreased over time (Supplementary Table S2, Figure 1).

### 3.2. Pharmacological Treatment

**Figure 2:**
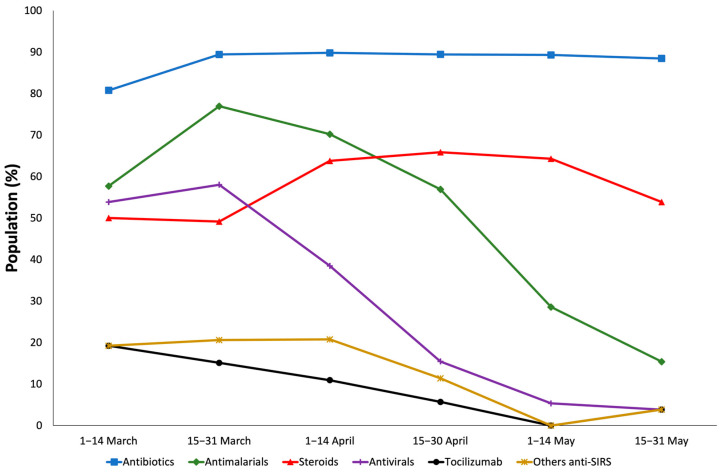
Trends in the use of the medicines used by in-hospital COVID-19 patients with severe acute respiratory syndrome in Castile and Leon (Spain) (1 March–31 May 2020).

With respect to medicines, antibiotics were the most used (89.27%), followed by antimalarials (68.10%), steroids (55.90%) antivirals (43.51%), tocilizumab (17.95%) and other anti-SIRS (11.9%) (Supplementary Table S2). Uses of antibiotics and steroids were constant over the study period, and uses of antimalarials, antivirals, tocilizumab and other anti-SIRS decreased (Supplementary Table S2, Figure 2).

There were no differences in drug use between deceased and non-deceased patients, except for steroids (*p* = 0.024) and tocilizumab (*p* = 0.001) which were more used by non-deceased, and anti-SIRS (*p* = 0.03) which were used more commonly by deceased patients (Table 3).

### 3.3. Survival and Risk Factor for Clinical Outcomes and Medication Prescribed

**Figure 3:**
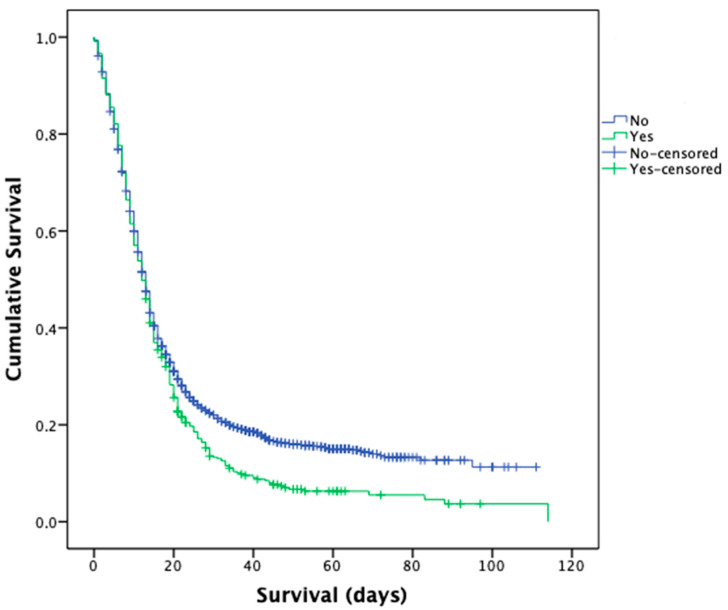
Kaplan–Meier survival curve for in-hospital COVID-19 patients with and without severe acute respiratory syndrome in Castile and Leon (Spain) (1 March–31 May 2020).

Survival of patients developing SARS was lower compared to patients without this complication (12 vs. 13 days, *p* = 0.001) (Figure 3). Main predictors of hospital death were: DIC (OR = 13.87), age (>65 years) (OR = 7.35), IMV (OR = 3.53), AKI (OR = 3.47), and cardiomyopathy (OR = 1.60). Anti-SIRS (OR = 2.08) and antivirals (OR = 1.80) were also related to death of the patients.

## 4. Discussion

According to the results emanated from this research, SARS was observed among one out of 10 hospitalized COVID-19 patients, which doubled the mortality rate observed among patients who did not develop SARS. Elderly males are the most common group to develop SARS, and have been related to an increased hospital stay and a greater requirement for ventilation. However, prevalence of this serious complication was dramatically lower compared to other regions in Spain, 14.03% vs. 31.5% [1] and 33.1% [2], even if the mean age in our population was greater than the age reported for other cohorts [19,20]. In any case, a mortality rate of 40% is consistent with mortality reported for cohorts outside of Spain [10] and lower compared to that reported for Spain [1,2]. Furthermore, as reported by others, hypertension, cardiovascular disease and diabetes are common companions of SARS [1,2]. The highest risk of hospital death has been reported in patients older than 65 years, who use anti-SIRS drugs and antivirals, who need IMV and who developed DIC, especially, and AKI.

From the beginning and still today, COVID-19-associated SARS is treated similarly to SARS of other etiologies [21]: low tidal volumes in association with ventilation in prone position, and the use of extracorporeal membrane is also a common recurse [22]. Our research show that IMV was used four times more than only conventional oxygen delivery. Indeed, as is well known, inflammation contributes to alter the permeability of the tissues engaged in gas exchange [23].

Importantly, between 1 March and 31 May 2020, most of the drugs were used off-label, as remdesivir was the only authorized medicine to treat COVID-19 [24]. Our research confirms to the real-world the benefits of other drugs in the treatment of COVID-19, tocilizumab, and steroids. Tocilizumab, an interleukin-6 (IL-6) receptor antagonist, acts against the “cascade” of pro-inflammatory cytokines, for which it has shown beneficial effects in the treatment of critically ill patients [25]. Steroids, such as methylprednisolone, also act against this “cascade” of cytokines, reducing systemic inflammation [26], especially in patients for whom tocilizumab previously failed [27,28]. In addition, methylprednisolone has been shown to be an effective treatment in hypoxemic hospitalized patients at high risk of acute respiratory failure (ARF), reducing mortality and improving markers of oxygenation and inflammation [29]. 

In any case, anti-SIRS and antivirals were probably associated with death because they were used in seriously ill patients. However, it would be highly desirable to conduct new clinical trials in relation to the efficacy and safety of these medicines in severe cases of COVID-19 [30].

Predictors of hospital stay were similar to that reported by other studies [1,31], highlighting DIC (OR:13.87), in order to sensitize clinicians on the nature of the infection that involves critical mechanisms of the immune system [9,23].

Some limitations should be mentioned. At the beginning and occasionally, the diagnosis of COVID-19 was performed according to clinical and radiological criteria without microbiological confirmation. Regarding pharmacological treatment, only recommendations from Spanish guidelines have been taken into account [17,18], so other possible treatments can be ignored in this analysis.

## 5. Conclusions

SARS is a very serious complication of COVID-19 with high morbidity and mortality. The clinical condition of hospitalized COVID-19 patients with SARS worsens in the population older than 65 years, who require IMV and develop DIC, AKI and cardiomyopathy, showing a higher probability of hospital death. Regarding pharmacological treatment, anti-SIRS and antiviral medicines are associated with a higher probability of death, possibly because their use is related to a more advanced stage of the disease and a worse prognosis. Furthermore, tocilizumab and steroids were associated with a low rate of hospital death, which confirm them today the main treatment for SARS in patients hospitalized for COVID-19.

Finally, the findings of this study, combined with those previously reported for other clinical conditions with bad prognosis in hospitalized COVID-19 patients (AKI [13] and previous CVD [14]), are decisive for the early detection of patients at risk, to determine the main predictors of mortality and to establish a protocol for clinical and pharmacological management based on previous experience.

## Figures and Tables

**Table 1:** Clinical criteria definition of SARS.

SARS	Temperature: >38 °C Early illness: equal to or more than two of the following: chills, rigors, myalgia, diarrhea, sore throat (self-reported or observed) Mild-to-moderate illness: indications of lower respiratory tract infection (cough, dyspnea) Severe illness: atypical pneumonia, presence of ARDS, autopsy findings in late patients.

Abbreviations: SARS, Severe acute respiratory syndrome, ARDS, acute respiratory distress syndrome.

**Table 2:** Baseline characteristics and clinical outcomes of in-hospital COVID-19 patients with severe acute respiratory syndrome in Castile and Leon (Spain) (1 March–31 May 2020).

		TOTAL	MALE	FEMALE	*p*
*N*		1025	650	375	0.004
Age (median and IQR)		78 (66–86)	76 (66–85)	81 (68–88)	0.004
Age < 65 (95% CI)		78.54 (76.02–81.05)	77.54 (74.33–80.75)	80.27 (76.24–84.29)	0.305
Age ≥ 65 (95% CI)		21.46 (18.95–23.98)	22.46 (19.25–25.67)	19.73 (15.71–23.76)	0.305
**Chronic Diseases (95% CI)**					
Hypertension		48.98 (45.92–52.04)	46.92 (43.09–50.76)	52.53 (47.48–57.59)	0.084
Cardiovascular disease		39.61 (36.62–42.6)	43.23 (39.42–47.04)	33.33 (28.56–38.1)	0.002
Diabetes		21.56 (19.04–24.08)	21.23 (18.09–24.37)	22.13 (17.93–26.34)	0.735
Chronic respiratory disease		16.49 (14.22–18.76)	18.15 (15.19–21.12)	13.6 (10.13–17.07)	0.058
Neoplasia		10.73 (8.84–12.63)	12.15 (9.64–14.67)	8.27 (5.48–11.05)	0.053
Autoimmune disease		7.9 (6.25–9.55)	9.08 (6.87–11.29)	5.87 (3.49–8.25)	0.066
Chronic Kidney disease		7.12 (5.55–8.7)	7.85 (5.78–9.91)	5.87 (3.49–8.25)	0.235
**Treatment**					
Oxygen delivery and ventilation (95% CI)					
IMV		16.68 (14.4–18.97)	20.15 (17.07–23.24)	10.67 (7.54–13.79)	0.001
Oxygen delivery		4.78 (3.47–6.09)	5.54 (3.78–7.3)	3.47 (1.62–5.32)	0.134
NIPPV		3.41 (2.3–4.53)	3.38 (1.99–4.77)	3.47 (1.62–5.32)	0.994
Medicines (95% CI)					
	Antibiotics	89.27 (87.37–91.16)	88.31 (85.84–90.78)	90.93 (88.03–93.84)	0.191
	Antimalarial	68.1 (65.24–70.95)	69.69 (66.16–73.23)	65.33 (60.52–70.15)	0.149
	Steroids	55.9 (52.86–58.94)	58.31 (54.52–62.1)	51.73 (46.68–56.79)	0.041
	Antivirals	43.51 (40.48–46.55)	47.08 (43.24–50.91)	37.33 (32.44–42.23)	0.002
	Tocilizumab	17.95 (15.6–20.3)	21.23 (18.09–24.37)	12.27 (8.95–15.59)	0.001
	Other anti-SIRS *	11.9 (9.92–13.88)	15.23 (12.47–17.99)	6.13 (3.7–8.56)	0.001
**Clinical Outcomes**					
Hospital LoS (median and IQR)		11 (6–19)	12 (6–21)	9 (5–16)	0.002
ICU LoS	(median and IQR)	15 (9–25)	15 (9–27)	15 (8–24)	0.403
	*N*	99	79	20	
Death (95% CI)		42.54 (39.51–45.56)	44.62 (40.79–48.44)	38.93 (34–43.87)	0.076
AKI (95% CI)		18.73 (16.34–21.12)	19.38 (16.35–22.42)	17.6 (13.75–21.45)	0.481
Fungal superinfection (95% CI)		5.37 (3.99–6.75)	6 (4.17–7.83)	4.27 (2.22–6.31)	0.236
Bacterial superinfection (95% CI)		4.39 (3.14–5.64)	5.23 (3.52–6.94)	2.93 (1.23–4.64)	0.084
SIRS (95% CI)		3.9 (2.72–5.09)	4.62 (3–6.23)	2.67 (1.04–4.3)	0.121
Cardiomyopathy (95% CI)		1.85 (1.03–2.68)	2.62 (1.39–3.84)	0.53 (0.2–1.27)	0.017
DIC (95% CI)		0.68 (0.18–1.19)	0.92 (0.19–1.66)	0.27 (0.06–0.59)	0.219

* Anakinra, baricitinib, interferón, ruxolitinib, siltuximab; Abbreviations: 95% CI, confidence interval, IQR, interquartile range, IMV, invasive mechanical ventilation, NIPPV, Noninvasive positive pressure ventilation, SIRS, systemic inflammatory response syndrome, LoS, length of stay, ICU, intensive care unit, AKI, acute kidney injury, DIC, disseminated intravascular coagulation.

**Table 3:** Medicines used by in-hospital COVID-19 patients with severe acute respiratory syndrome in Castile and Leon (Spain) (1 March–31 May 2020).

Medicines	Total (95% CI)	Death (95% CI)	No-Death (95% CI)	*p*
	*N* = 1025	*N* = 589	*N* = 436	
Antibiotics	89.27 (87.37–91.16)	88.62 (86.06–91.19)	90.14 (87.34–92.94)	0.439
Ceftriaxone	67.51 (64.65–70.38)	66.38 (62.57–70.2)	69.04 (64.7–73.38)	0.37
Azithromycin	65.37 (62.45–68.28)	69.61 (65.9–73.32)	59.63 (55.03–64.24)	0.001
Levofloxacin	22.34 (19.79–24.89)	19.19 (16.01–22.36)	26.61 (22.46–30.75)	0.005
Teicoplanine	1.27 (0.58–1.95)	1.19 (0.31–2.06)	1.38 (0.28–2.47)	0.791
Cefditoren	4.68 (3.39–5.98)	5.77 (3.89–7.66)	3.21 (1.56–4.87)	0.055
Clarithromycin	0.78 (0.24–1.32)	0.68 (0.02–1.34)	0.92 (0.02–1.81)	0.668
Moxifloxacin	0.2 (0.08–0.39)	0 (0–0)	0.46 (0.18–0.85)	0.1
Cefotaxime	0.2 (0.08–0.39)	0.17 (0.06–0.41)	0.23 (0.02–0.56)	0.831
Ceftaroline	0 (0–0)	0 (0–0)	0 (0–0)	-
Antimalarials	68.1 (65.24–70.95)	66.55 (62.74–70.36)	70.18 (65.89–74.48)	0.218
Hydroxychloroquine	63.8 (60.86–66.75)	63.5 (59.61–67.39)	64.22 (59.72–68.72)	0.812
Chloroquine	5.27 (3.9–6.64)	3.74 (2.2–5.27)	7.34 (4.89–9.79)	0.011
Steroids	55.9 (52.86–58.94)	58.91 (54.94–62.89)	51.83 (47.14–56.52)	0.024
Methylprednisolone	54.05 (51–57.1)	56.54 (52.53–60.54)	50.69 (46–55.38)	0.063
Prednisone	10.83 (8.93–12.73)	13.58 (10.82–16.35)	7.11 (4.7–9.52)	0.001
Antivirals	43.51 (40.48–46.55)	41.94 (37.95–45.92)	45.64 (40.97–50.32)	0.237
Lopinavir-Ritonavir	43.41 (40.38–46.45)	41.94 (37.95–45.92)	45.41 (40.74–50.09)	0.267
Remdesevir	0.29 (0.04–0.54)	0.17 (0.06–0.41)	0.46 (0.18–0.85)	0.397
Tocilizumab	17.95 (15.6–20.3)	23.26 (19.85–26.67)	10.78 (7.87–13.69)	0.001
Other anti-SIRS	11.9 (9.92–13.88)	10.02 (7.59–12.44)	14.45 (11.15–17.75)	0.03
Interferon Beta	8.98 (7.23–10.73)	7.3 (5.2–9.4)	11.24 (8.27–14.2)	0.029
Anakinra	2.54 (1.57–3.5)	2.72 (1.4–4.03)	2.29 (0.89–3.7)	0.67
Ruxolitinib	0.1 (0.09–0.12)	0 (0–0)	0.23 (0.02–0.56)	0.245
Baricitinib	0.59 (0.12–1.05)	0.51 (0.07–0.92)	0.69 (0.09–1.35)	0.711

Abbreviations: 95% CI, confidence interval, SIRS, systemic inflammatory response syndrome.

## Data Availability

Restrictions apply to the availability of these data. Data were obtained from regional health authorities (Gerencia Regional de Salud (GRS)) and may be requested from sdinvestigacion@saludcastillayleon.es (GRS).

## Supplementary Materials

The following supporting information can be downloaded at: https://www.mdpi.com/article/10.3390/medicina58060829/s1, Table S1: List of medicines used in the COVID-19 treatment according to Spanish guidelines; Table S2: Treatment and clinical outcomes evolution of in-hospital COVID-19 patients with acute respiratory distress syndrome in Castile and Leon (Spain) (1 March–31 May 2020).
