# Supplementary material for: Hospitalized COVID-19 Patients with Severe Acute Respiratory Syndrome: A Population-Based Registry Analysis to Assess Clinical Findings, Pharmacological Treatment and Survival
Source: Medicina (Kaunas). 2022 Jun 19;58(6):829. doi: 10.3390/medicina58060829 (PMC9230059; doi:10.3390/medicina58060829)
Supplement: Supplementary file 1 [file medicina-58-00829-s001.zip › medicina-1770341-supplementary.pdf]

Supplementary Table S1. List of medicines used in the COVID-19 treatment according to Spanish guidelines [11,12].

Medicines Type	ATC Code	Medicine	Medicines Type	ATC Code	Medicine
Antibiotics	J01DD01	Cefotaxime	Anti SIRS Drugs	L01XE18	Ruxolitinib
	J01DD04	Ceftriaxone		L03AB05	Interferon alpha 2b
	J01DD16	Cefditoren		L03AB08	Interferon beta 1b
	J01DI02	Ceftaroline		L04AA37	Baricitinib
	J01FA09	Clarithromycin		L04AC03	Anakinra
	J01FA10	Azithromycin		L04AC07	Tocilizumab
	J01MA12	Levofloxacin		L04AC11	Siltuximab
	J01MA14	Moxifloxacin		L04AC14	Sarilumab
	J01XA02	Teicoplanine	Antivirals	J05AR10	Lopinavir and Ritonavir
Antimalarials	P01BA01	Chloroquine		J05AX95*	Remdesivir
	P01BA02	Hidroxychloroquine	Steroids	H02AB04	Methylprednisolone
				H02AB07	Prednisone

*Provisional ATC Code

Abbreviations: SIRS, systemic inflammatory response syndrome

Supplementary Table S2. Treatment and clinical outcomes evolution of in-hospital COVID-19 patients with acute respiratory distress syndrome in Castile and Leon (Spain) (March 1st - May 31st, 2020).

	**1-14 March**	**15-31 March**	**1-14 April**	**15-30 April**	**1-14 May**	**15-31 May**
N	26	529	265	123	56	26
**Antibiotics**	**80.77 (65.62-95.92)**	**89.41 (86.79-92.04)**	**89.81 (86.17-93.45)**	**89.43 (84-94.86)**	**89.29 (81.18-97.39)**	**88.46 (76.18-100.74)**
Ceftriaxone	46.15 (26.99-65.32)	67.67 (63.69-71.66)	68.3 (62.7-73.9)	65.04 (56.61-73.47)	78.57 (67.82-89.32)	65.38 (47.1-83.67)
Azithromycin	26.92 (9.87-43.97)	65.6 (61.55-69.64)	70.57 (65.08-76.05)	66.67 (58.34-75)	60.71 (47.92-73.51)	50 (30.78-69.22)
Lovofloxacin	50 (30.78-69.22)	23.06 (19.47-26.65)	18.87 (14.16-23.58)	21.95 (14.64-29.27)	17.86 (7.83-27.89)	26.92 (9.87-43.97)
Cefditoren	-	5.48 (3.54-7.42)	3.77 (1.48-6.07)	4.88 (1.07-8.68)	3.57 (0.29-5.43)	3.85 (0.55-8.24)
Clarithromycin	3.85 (0.55-8.24)	0.57 (0.07-1.03)	0.38 (0.06-0.94)	1.63 (0.61-2.86)	1.79 (0.68-3.25)	-
Teicoplanin	3.85 (0.55-8.24)	1.13 (0.23-2.04)	2.26 (0.47-4.06)	-	-	-
Cefotaxime	-	0.19 (0.08-0.36)	0.38 (0.06-0.94)	-	-	-
Moxifloxacin	-	-	0.75 (0.29-1.62)	-	-	-
Ceftaroline	-	-	-	-	-	-
**Antimalarials**	**57.69 (38.7-76.68)**	**76.94 (73.35-80.53)**	**70.19 (64.68-75.7)**	**56.91 (48.16-65.66)**	**28.57 (16.74-40.4)**	**15.38 (1.52-29.25)**
Hydroxychloroquine	46.15 (26.99-65.32)	70.7 (66.82-74.58)	68.3 (62.7-73.9)	56.1 (47.33-64.87)	26.79 (15.19-38.38)	11.54 (0.74-22.82)
Chloroquine	11.54 (0.74-22.82)	8.13 (5.8-10.46)	1.89 (0.25-3.52)	0.81 (0.07-2.24)	1.79 (0.68-3.25)	3.85 (0.55-8.24)
**Steroids**	**50 (30.78-69.22)**	**49.15 (44.89-53.41)**	**63.77 (57.99-69.56)**	**65.85 (57.47-74.23)**	**64.29 (51.74-76.84)**	**53.85 (34.68-73.01)**
Methylprednisolone	46.15 (26.99-65.32)	47.64 (43.38-51.89)	61.51 (55.65-67.37)	63.41 (54.9-71.93)	64.29 (51.74-76.84)	50 (30.78-69.22)
Prednisone	11.54 (0.74-22.82)	10.02 (7.46-12.58)	12.45 (8.48-16.43)	13.01 (7.06-18.95)	5.36 (0.54-10.25)	11.54 (0.74-22.82)
**Antivirals**	**53.85 (34.68-73.01)**	**58.03 (53.83-62.24)**	**38.49 (32.63-44.35)**	**15.45 (9.06-21.83)**	**5.36 (0.54-10.25)**	**3.85 (0.55-8.24)**
Lopinavir-Ritonavir	53.85 (34.68-73.01)	58.03 (53.83-62.24)	38.11 (32.27-43.96)	15.45 (9.06-21.83)	5.36 (0.54-10.25)	3.85 (0.55-8.24)
Remdesevir	0 (0-0)	0.38 (0.14-0.76)	0.38 (0.06-0.94)	-	-	-
**Tocilizumab**	**19.23 (4.08-34.38)**	**20.6 (17.16-24.05)**	**20.75 (15.87-25.64)**	**11.38 (5.77-16.99)**	**-**	**3.85 (0.55-8.24)**
**Other Anti-SIRS**	**19.23 (4.08-34.38)**	**15.12 (12.07-18.18)**	**10.94 (7.18-14.7)**	**5.69 (1.6-9.79)**	**-**	**3.85 (0.55-8.24)**
Interferon Beta	19.23 (4.08-34.38)	13.61 (10.69-16.53)	5.28 (2.59-7.98)	-	-	3.85 (0.55-8.24)
Anakinra	-	1.51 (0.47-2.55)	4.53 (2.02-7.03)	4.07 (0.58-7.55)	-	3.85 (0.55-8.24)
Baricitinib	-	0.19 (0.08-0.36)	1.13 (0.14-2.16)	1.63 (0.61-2.86)	-	-
Ruxolitinib	-	0.19 (0.08-0.36)	-	-	-	-
Hospital LoS (median + IQR)	18.5 (8-42)	11 (6-21)	12 (5-19)	10 (6-16)	8 (5-13.5)	5.5 (4-10)
ICU LoS (median + IQR)	23	15.50(9-26)	13 (8-22)	18 (8-22)	5.5 (1-10)	-
	N=1	N=71	N=19	N=4	N=4	N=0
Death (% 95 CI)	76.92 (60.73-93.12)	48.58 (44.32-52.84)	38.49 (32.63-44.35)	30.08 (21.98-38.19)	26.79 (15.19-38.38)	19.23 (4.08-34.38)
AKI (% 95 CI)	3.85 (0.55-8.24)	18.34 (15.04-21.63)	20.38 (15.53-25.23)	19.51 (12.51-26.52)	19.64 (9.24-30.05)	19.23 (4.08-34.38)
Fungal superinfection (% 95 CI)	15.38 (1.52-29.25)	5.48 (3.54-7.42)	3.77 (1.48-6.07)	8.94 (3.9-13.99)	1.79 (0.68-3.25)	-
Bacterial superinfection (% 95 CI)	3.85 (0.55-8.24)	3.21 (1.71-4.72)	6.04 (3.17-8.91)	2.44 (0.29-4.17)	10.71 (2.61-18.82)	7.69 (2.55-13.93)
SIRS (% 95 CI)	3.85 (0.55-8.24)	2.84 (1.42-4.25)	5.66 (2.88-8.44)	3.25 (0.12-6.39)	5.36 (0.54-10.25)	7.69 (2.55-13.93)
Carodiomyopathy (% 95 CI)	-	1.51 (0.47-2.55)	1.13 (0.14-2.16)	4.88 (1.07-8.68)	1.79 (0.68-3.25)	3.85 (0.55-8.24)
DIC (% 95 CI)	-	0.57 (0.07-1.03)	0.75 (0.29-1.62)	-	1.79 (0.68-3.25)	3.85 (0.55-8.24)

Abbreviations: 95 CI, confidence interval, LoS, length of stay, ICU, intensive care unit, AKI, acute kidney injury, SIRS, systemic inflammatory response syndrome, DIC, disseminated intravascular coagulation.
